# Role of telitacicept in the treatment of IgA nephropathy

**DOI:** 10.1186/s40001-023-01320-2

**Published:** 2023-09-22

**Authors:** Lijun Wu, Xinru Du, Xuehong Lu

**Affiliations:** The Second Hospital of Jilin University Nephropathy of Internal Medicine, Changchun, 130022 China

**Keywords:** APRIL, BAFF, BLyS, IgA, Nephropathy, Telitacicept

## Abstract

IgA nephropathy (IgAN) is the most common primary glomerular disease in the world, and up to 40% of patients with IgAN develop end-stage renal disease (ESRD). At present, an increasing amount of evidence indicates that the pathogenesis of IgAN is related to autoimmunity. In recent years, several studies have shown that B cell activating factors (BAFF), also known as B lymphocyte stimulators (BLyS), and proliferation-inducing ligand APRIL are extremely important for the activation of autoimmune signalling pathways, which have become key targets for the treatment of IgAN. As a dual-target biological agent, telitacicept can inhibit both BLyS and APRIL cytokines, improve the function of renal immune complexes, and reduce haematuria and proteinuria, which play important roles in IgAN pathogenesis and long-term prognosis. This article reviews the role of telitacicept in IgA nephropathy and discusses its potential for use in the treatment of IgAN and other autoimmune diseases where pathogenesis is driven by B cells.

## Introduction

The most prevalent primary glomerular disease in the world is IgA nephropathy (IgAN). In IgAN, immunoglobulin A (IgA) is deposited in the mesangial region of the glomeruli, which causes damage to the mesangial region [1, 2]. There are between 5.80 and 8.23 million IgAN patients in China, with around 2.5 cases per 100,000 people. Approximately 20–40% of IgAN patients in China go on to develop end-stage renal disease (ESRD) [3, 4], and the need for renal replacement therapy places a significant financial burden on their families and on society as a whole [5, 6].

The pathophysiology of IgAN is poorly understood; however, haematuria, proteinuria, and progressive renal failure are the most prominent clinical signs. Angiotensin-converting-enzyme inhibitors (ACEI), also known as angiotensin II-receptor blockers (ARB), are being used as supportive therapy for IgAN to manage blood pressure and reduce urine protein. A conservative supportive therapy of renin–angiotensin–aldosterone system (RAAS) blockers has also been advised (Kidney Disease: Improving Global Outcomes, KDIGO) [7]. However, some patients cannot tolerate these drugs as they experience decreased renal function and so cannot reach the maximum dose. The side effects of hormones and immunosuppressants can also prevent long-term use [8]. Thus, current therapies do not adequately treat progressive IgAN, and novel approaches are needed.

Telitacicept (Taiai), also known as RC18 or RCT18, is a fully human soluble fusion protein. It contains the crystalline fragment (Fc) of human immunoglobulin G1 (IgG1) combined with the extracellular specific soluble portion of the trans-membrane activator and CAML-interactor (TACI) of the B lymphocyte stimulator (BLyS), also known as B cell activating factor (BAFF) [9, 10]. Telitacicept is a novel biological agent that targets two kinds of cell signalling molecules essential for B lymphocyte development. The interaction of the BLyS and a proliferation-inducing ligand (APRIL) can successfully suppress the B cell-mediated autoimmune response [11]. According to a current clinical trial, telitacicept dramatically reduces proteinuria while displaying a good safety profile [12].

By examining the pathophysiology and treatment options associated with telitacicept, we analysed its role in IgA nephropathy and proposed new approaches for the treatment of IgAN in the future.

## Discussion

### Role of B lymphocytes in the pathogenesis of IgAN

Activation of B lymphocytes plays a crucial role in autoimmunity, which is directly associated with the early stages of IgAN. According to the traditional “Four hits” hypothesis, the first hit is described as follows: under the influence of potential environmental and genetic factors, the circulating level of galactose-deficient IgA1 (Gd-IgA1) increases; the second hit is the production of Gd-IgA1-specific autoantibody IgG; the third hit is the presence of Gd-IgA1 in the circulating immune complex; and the fourth hit is that the Gd-IgA1–antiglycan IgG immunological complex deposits in the mesangium, causing inflammation and fibrosis in the mesangial region of the glomerulus [13, 14].The first hit is the production of Gd-IgA1 by activated B cells as a result of mis-homing [15]. The immunoglobulin mucosal-associated lymphoid tissue (MALT) mainly produces and secretes IgA [16]. IgAN is believed to be most commonly linked to the formation of gut-associated lymphoid tissue (GALT) and nasopharynx-associated lymphoid tissue (NALT) [17, 18]. Through T cell-dependent or independent mechanisms, pathogenic bacteria in these areas promote the maturation and differentiation of immature B lymphocytes, which in turn produce plasma cells that secrete IgA [19]. Additionally, dysregulation of the gut microbiota has been linked to the development of GALT; it may also be able to increase BAFF expression and dendritic cell maturation by activating Toll-like receptors (TLRs), promote B lymphocyte maturation and plasma cell production, and accelerate the development of IgAN [20].

A range of cytokines, including BLyS/BAFF and APRIL, control the activation of B cells [21, 22]. BLyS/BAFF is a crucial B lymphocyte activation factor. These cytokines bind to TACI, the BAFF receptor (BAFF-R), or the B cell maturation antigen (BCMA) to induce B lymphocyte maturation and differentiation [11, 23, 24]. According to clinically controlled experimental studies, the density of glomerular mesangial IgA deposition and levels of serum IgA1 are positively correlated with serum levels of BLyS/BAFF levels in IgAN patients. This suggests that elevated BLyS/BAFF levels can lead to excessive IgA1 production and accelerate the progression of IgAN [25]. Furthermore, Mccarthy DD et al. [26] discovered that serum IgA levels were raised in transgenic mice that overexpressed BAFF. The mice displayed symptoms of autoimmune illnesses, and glomerular mesangial IgA deposition could be clearly seen. APRIL is a cytokine that resembles tumour necrosis factor (TNF) and shares 30% of its sequence with BLyS/BAFF. It has a high binding affinity for BCMA and TACI and helps mature B cells differentiate into plasma cells and maintain plasma cells [11, 27, 28]. Zhang et al. demonstrated that the plasma APRIL levels of IgAN patients were significantly higher than in the healthy control group, and that there was a significant positive correlation between the plasma APRIL level and the adjusted Gd-IgA1 level [29]. A previous study by Muto et al [30] also described a marked increase in APRIL expression in the tonsils of patients with IgAN, a positive response to tonsillectomy in patients with excessive APRIL expression, and a decrease in serum Gd-IgA1 levels. This supports our theory that APRIL plays a role in controlling IgAN progression. Thus, inhibiting B lymphocyte activation, lowering IgA levels, and targeting BLyS/BAFF and APRIL are emerging strategies for the investigation of IgAN therapeutic medicines [31].

### Treatment of IgAN

#### Clinical treatment goals

Although the underlying pathophysiology of IgAN has been extensively investigated, we still lack precise therapies for the condition. Currently, IgAN is treated through therapeutically supportive measures to lower blood pressure, lessen proteinuria, reduce lifestyle risk factors, and lessen other non-specific kidney damage. The main approach to intervention is the use of ACEI or ARB, which lower intraglomerular pressure by inhibiting the RAAS, to some extent lowering the patient's proteinuria levels, and delaying the progression of renal failure [32].

#### Traditional treatment modalities

If IgAN patients have concurrent hypertension and the patient’s proteinuria is greater than 1.5 g/day, 2021 KDIGO advises stratified management with RAAS blockade therapy; if the patient’s urine protein remains between 0.75 and 1 g/day after 3 months of optimised supportive therapy, 6 months of glucocorticoid therapy is recommended [33].

However, the effectiveness of glucocorticoids and immunosuppressants in patients with IgAN is still debatable. According to research by the supportive versus immunosuppressive therapy for the treatment of progressive IgA nephropathy (STOP-IgAN) [34], immunosuppressive regimens do not significantly enhance prognosis in relation to that by supportive therapy. Additionally, they have increased negative side effects, such as the development of infection and cancer.

Use of traditional Chinese medicine has also shown results in IgAN [35]. The dialectical treatment of traditional Chinese medicine is effective for IgAN patients with primary chronic lesions (tubular atrophy and renal small blood vessel lesions), whereas IgAN patients with active lesions and chronic kidney lesions require integration of traditional Chinese and Western medicine.

#### Novel biologics

In recent years, several innovative biologics have started to be employed in the treatment of IgAN as a result of advances in our understanding of the disease's aetiology. Sparsentan is a non-immunosuppressive, single-molecule, dual endothelin and angiotensin receptor antagonist (DEARA), with a double effect as a highly selective antagonist of endothelin type A (ETA) receptor and angiotensin II type-1 receptors (AT1R). An ongoing phase III clinical trial (NCT03762850), one of the largest intervention experiments to study the efficacy and safety of new IgAN drugs, is studying the efficacy and safety of Sparsentan (400 mg once daily) compared with the current standard therapy irbesartan (300 mg once daily) in the treatment of IgAN. A pre-specified interim analysis of the primary efficacy endpoint of 24-h urine proteinuria change in subjects is performed at week 36. And the study met this efficacy endpoint, and the results were statistically significant: after 36 weeks of treatment, the proteinuria level of patients in the sparsentan group was reduced by an average of 49.8% from baseline. This decrease was more than three times higher than that of the irbesartan-treated group, i.e. the positive drug control group (49.8% vs 15.1%; *p* < 0.0001), and the safety profile was similar between the two groups [36–38]. Drugs that target the B cell receptor, like CD20 monoclonal antibodies, work in a number of ways, including cytotoxicity-dependent, complement-dependent, and apoptosis-inducing pathways [39]. Rituximab is a CD20 monoclonal antibody. However, according to a randomised controlled trial by Richard A et al [40], it did not lower serum levels of Gd-IgA1 and glycan-specific IgG antibodies, enhance renal function, or reduce proteinuria. Rituximab is a mild anti-CD20 monoclonal antibody that causes B lymphocyte failure by binding to CD20 receptors on the membrane of pre-B lymphocytes and mature B lymphocytes [41]. However, because plasma cell membranes lack CD20 receptors, rituximab cannot bind to plasma cells or clear plasma cells, and may therefore ineffectively inhibit plasma cell formation and the secretion of Gd-IgA1 antibodies. This could be the cause of rituximab's inability to significantly alter the course of treatment or prognosis for IgAN. In a single-arm trial, 24 patients with primary glomerulonephritis—five of whom had IgAN—were treated with a single dose of rituximab at 375 mg/m^2^. In patients with IgAN, proteinuria did not significantly alter after 6 months of rituximab treatment (1.0 ± 0.8 g/day at baseline vs 0.9 ± 0.8 g/day at 6 months). The short follow-up of just 6 months and the potential need for many doses of rituximab to produce a response in a slow-progressing condition like IgAN made it difficult to interpret the trial's findings [42, 43]. A fully human monoclonal antibody to BLyS(BAFF), belimumab reduces immature and mature B lymphocytes directly and indirectly by inhibiting the function of plasma cells [44], which may explain why it is ineffective at preventing the production of Gd-IgA1 antibodies in plasma cells. It also may explain why belimumab is ineffective at improving the effectiveness and prognosis of IgAN. Narsoplimab (OMS721) is a human monoclonal antibody targeting mannan-binding lectin-associated serine protease-2 (MASP-2), the effector enzyme of the lectin pathway of the complement system [45]. OMS721 is being developed for treatment of diseases thought to be mediated by the complement lectin pathway, including IgAN [46]. Research is ongoing in a phase III, double-blind, randomised, placebo-controlled experiment (NCT03608033) in which 450 patients have been randomly divided into 2 groups, treated with OMS721 at 185 mg/mL, and controlled with 5% glucose (D5W) or normal saline in the placebo group. Observations include changes from the baseline in a 24-h urine protein excretion (UPE) in g/day at 36 weeks from the beginning of the treatment. Results of this experiment are expected to open up new options for IgAN treatments. There are many studies on IgAN treatments conducted globally [46], and the list summarises the treatments for IgAN mentioned in this article (Table 1) [44, 46].

**Table 1:** Therapies in clinical development for treatment of IgAN

Agent	Target	Format	Mechanism of action	Stage in IgAN to date	References
Rituximab	CD20	Monoclonal antibody	Depletes CD20 B cells	Phase 4	NCT00498368
Belimumab	BAFF	Monoclonal antibody	Inhibits activation of B cells	None	44
Narsoplimab	MASP-2	Monoclonal antibody	Inhibits complement lectin pathway activation	Phase 3	NCT03608033
Telitacicept	BAFF and APRIL	Fusion protein/antibody	Inhibits maturation and activation of B cells	Phase 2	NCT04291781
Atacicept	BAFF and APRIL	Fusion protein/antibody	Inhibits maturation and activation of B cells	Phase 3	NCT04716231
Sparsentan	ETAR and AT1R	Non-immunosuppressive, single-molecule	Vasodilator effects	Phase 3	NCT03762850

As a result, new therapeutic targets need to be considered when designing pharmacological treatments. New therapeutic targets must successfully combat re-B lymphocytes, mature B lymphocytes, plasma cells, and the formation of Gd-IgA1 antibodies. This objective can now be accomplished by a novel biologic, which offers fresh start for the treatment, prognosis, and long-term outcome of IgAN.

### Role of telitacicept in the treatment of IgAN

#### Mechanism of action of telitacicept

**Fig. 1:**
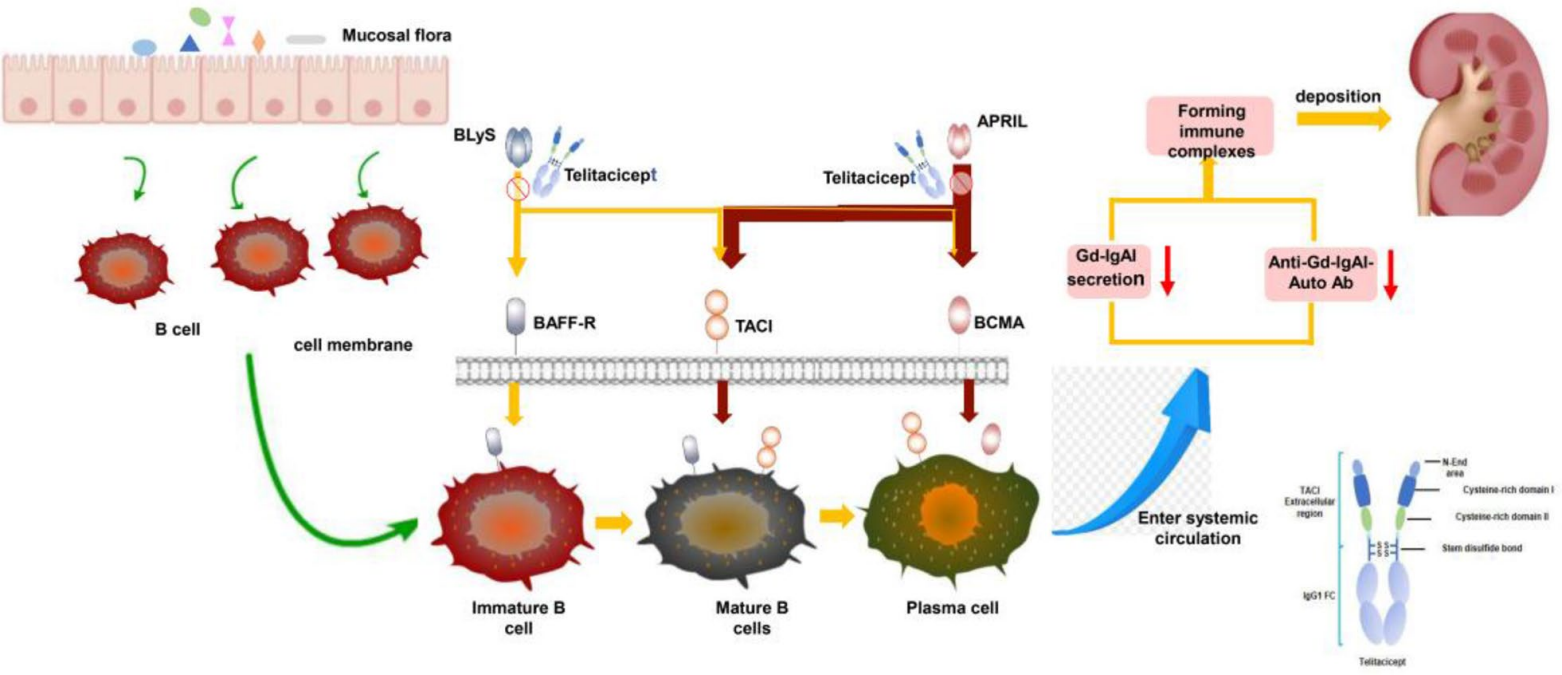
Mechanism of action of telitacicept in the treatment of IgAN. Immature B lymphocytes are inappropriately activated by mucosal flora in the mucosal epithelial region. By binding two important cytokines, the B lymphocyte stimulator (BLyS) and a proliferation-inducing ligand (APRIL), with a B cell activating factor receptor (BAFF-R), the trans-membrane activator and CAML-interactor (TACI), and the B cell maturation antigen (BCMA), the three B lymphocyte-membrane-surface-related receptors, abnormally activate immature B lymphocytes in bone marrow and peripheral lymphoid tissues mature and differentiate. The mature differentiated plasma cells enter the systemic circulation, galactose-deficient IgA1 (Gd-IgA1) secretion increases, anti-galactose-deficient IgA1 (anti-Gd-IgA1) autoantibody secretion increases, and a more immune complex forms. As a biological agent can simultaneously block cytokines BLys and APRIL, it can also prevent their binding to the three B lymphocyte-membrane-surface-related receptors (BAFF-R, TACI, and BCMA). This prevents the maturation of B lymphocytes, thereby preventing the release and development of Gd-IgA1 and anti-Gd-IgA1 antibodies into the bloodstream and preventing the development of immunological complexes in the kidney's mesangial region. Telitacicept is a fusion protein comprising a recombinant trans-membrane activator, calcium modulator, and cyclophilin ligand interactor (TACI) receptor fused to the fragment crystallisable (Fc) domain of human immunoglobulin G (IgG)

Telitacicept is an innovative medicine that was independently developed in China and has the qualities of “a new target, a new structure, and a new mechanism” for the treatment of autoimmune diseases [9]. The dual-target action of telitacicept can more successfully lower B lymphocyte activation, limit the body’s immune response, and treat autoimmune illnesses than belimumab, an inhibitor of BLyS/BAFF [11]. Additionally, telitacicept has a slightly higher molecular weight than the identical BLyS/BAFF and APRIL dual-target inhibitor, atacicept (NCT04716231) (TACI-Ig) (73.4 kD vs. 80.24 kD). This helps it to maintain the N-terminal portion of extracellular TACI and enhances its capacity to bind to TACI [47]. Previous studies have indicated that atacicept can, in a dose-dependent manner, lower serum levels of Gd-IgA1 and anti-Gd-IgA1 antibodies in IgAN patients [48, 49]. Since telitacicept targets both BLyS/BAFF and APRIL, it has potential use in the treatment of many autoimmune illnesses linked to B cells such as IgAN, systemic lupus erythematosus (SLE), and rheumatoid arthritis (RA) [25, 50]. Blocking the binding of BLyS/BAFF to TACI and BAFF-R effectively slows down the maturation and differentiation of B lymphocytes. In addition to this, by blocking the binding of APRIL to BCMA or TACI, telitacicept can lower IgA1 secretion, lower the production of Gd-IgA1, and inhibit the production of anti-Gd-IgA1 autoantibodies. This slows the progression of IgAN by reducing immune complex deposition in the mesangial region of the glomeruli (Fig. 1) [23, 51–56].

#### Clinical efficacy

Based on a recent phase II clinical trial (NCT04291781), after 24 weeks of continuous administration of telitacicept at 240 mg/week, patients’ average 24-h proteinuria levels decreased by 49% from baseline 24-h proteinuria. After 24 weeks of continuous administration at 160 mg/week or 240 mg/week, the estimated glomerular filtration rate (eGFR) increased significantly in relation to that of the placebo group, and the patients' serum levels of IgA, IgG, and IgM dramatically dropped [12]. Larger, high-quality clinical investigations are required to further confirm the effectiveness of telitacicept against IgAN.

In our country, telitacicept is currently approved for the treatment of SLE [11], and clinical research is being conducted on its potential use against multiple sclerosis (MS), neuromyelitis optica spectrum disorder (NMOSD), myasthenia gravis (MG), and primary Sjogren's syndrome (pSS) [10, 57]. Thus far, telitacicept has shown substantial promise in the treatment of B lymphocyte-associated immunological disorders as numerous clinical trials have progressed.

#### Clinical monitoring indicators related to IgAN were established

Despite the lack of defined indicators for IgAN clinical monitoring, immune variables, inflammatory factors, and indicators related to renal function are frequently utilised to assess clinical efficacy.

In recent years, the serum IgA/C4 ratio, serum soluble IL-2 receptors (IL-2R) level [58], urine type IV collagen content [59], Monocyte chemoattractant protein 1(MCP-1) level [60], and urinary IL-6 levels [61] have all been found to be relevant to IgAN progression and can serve as potential new indicators in the clinical monitoring of IgAN.

Xie et al. [62] discovered that the intensity of glomerular CD206 + and CD68 + macrophage infiltration in IgAN patients predicted the patient's response to immunosuppression; When the level of glomerular CD206 + macrophage infiltration was increased, the body's response to immunosuppression increased by a factor of 40; and when the level of glomerular CD68 + macrophages infiltration was increased, the body’s response rate to immunosuppression increased by a factor of 13. Therefore, clinicians can develop individualised IgAN treatment regimens based on the degree of glomerular CD206 + and CD68 + macrophage infiltration.

In addition to understanding the mechanism of therapeutic intervention, evaluation of the clinical efficacy of IgA therapy requires examination of both the illness characteristics and the function of the injured organs. Telitacicept is a two-target immunosuppressant, and for patients treated with telitacicept, we may be able to monitor treatment and prognosis with CD206 and CD68.

#### Safety of telitacicept

Telitacicept has fewer side effects than conventional medications since it is processed by cells rather than the liver and kidneys [9]. The side effects seen in the placebo group were similar to those experienced by the telitacicept group. Importantly, in phase II randomised, double-blind, placebo-controlled clinical trials of telitacicept in 44 patients with IgAN and persistent proteinuria, all observed side effects were considered mild to moderate [12]. Additionally, there was no increase in the frequency of adverse reactions in patients when the dose was increased from 160 mg/week to 240 mg/week.

Telitacicept has also shown good tolerance and safety in the treatment of patients with SLE [63] and RA [64]. In this 52-week phase III, placebo-controlled, multicenter, randomised, double-blind clinical trial (NCT04082416), the efficacy and safety of telitacicept (160 mg subcutaneously once weekly) were compared with those of a placebo (subcutaneously once weekly) in the treatment of SLE. The SLE Response Index 4 (SRI-4) response rates in the telitacicept and placebo groups were evaluated at week 52 as the primary endpoint. The SRI-4 response was defined as the SELENA-SLEDAI score being reduced by ≥ 4 points from baseline, no new BILAG assessment class A organs or ≤ 2 new BILAG assessment B organs compared to baseline, and the physician's overall assessment (PGA) not deteriorating (< 0.3 points increase from baseline) at 52 weeks. The SRI-4 response rate in the telitacicept group (160 mg subcutaneously once weekly) reached 82.6%, significantly higher than that in the placebo group of 38.1%, (82.6% vs 38.1%; *p* < 0.001). In addition, the overall infection rate was similar between the telitacicept and placebo groups, with no significant difference, suggesting the good tolerability and safety profile of telitacicept. In a recently published case series, telitacicept was used to treat refractory childhood SLE; The course of treatment lasted 5–26 weeks, and 4 out of 15 patients experienced mild-to-moderate side effects [65]. According to the studies mentioned above, telitacicept is safe and effective for use in the treatment of a range of autoimmune diseases, including IgAN.

#### Epilogue

IgAN is the most prevalent form of primary glomerulonephritis in the world, including in China [55]. Between 20% and 40% of patients develop ESRD within 20 years of IgAN, putting both patients and their families under significant physical and financial strain. The development of telitacicept provides new possibilities for the treatment and long-term prognosis of IgAN, and for other autoimmune diseases where B cells play a primary role in pathogenesis. The safety and efficacy of telitacicept remain to be further confirmed as clinical trials progress.

## Data Availability

Data sharing is not applicable to this article as no datasets were generated or analysed during the current study.
